# ﻿Description of two new species of *Psilochasmus* Lühe, 1909 (Digenea, Psilostomidae), with remarks on the diversity of the genus and a key to its species

**DOI:** 10.3897/zookeys.1254.162728

**Published:** 2025-10-01

**Authors:** Tyler J. Achatz, Lauren B. Morton, Sarah A. Orlofske, Sara V. Brant, Martin M. Montes, Federico Bondone, Vasyl V. Tkach

**Affiliations:** 1 Department of Natural Sciences, Middle Georgia State University, Macon, Georgia, USA Middle Georgia State University Macon United States of America; 2 Department of Biology and Olson Museum of Natural History, University of Wisconsin – Stevens Point, Stevens Point, Wisconsin, USA University of Wisconsin – Stevens Point Stevens Point United States of America; 3 Museum of Southwestern Biology Division of Parasites, Department of Biology, University of New Mexico, Albuquerque, New Mexico, USA University of New Mexico Albuquerque United States of America; 4 Centro de Estudios Parasitológicos y Vectores (CEPAVE), Consejo Nacional de Investigaciones Científicas y Técnicas, Universidad Nacional de La Plata (CCT, CONICET-UNLP), La Plata, Buenos Aires, Argentina Universidad Nacional de La Plata (CCT, CONICET-UNLP) La Plata Argentina; 5 Centro de Rescate de Fauna Silvestre – Ecoparque, Ciudad Autónoma de Buenos Aires, Buenos Aires, Argentina Centro de Rescate de Fauna Silvestre - Ecoparque Buenos Aires Argentina; 6 Department of Biology, University of North Dakota, Grand Forks, North Dakota, USA University of North Dakota Grand Forks United States of America

**Keywords:** Molecular phylogeny, *Psilochasmus oxyurus*, *Psilochasmus slavaukrainii* sp. nov., *Psilochasmus urbeni* sp. nov., Psilostomidae

## Abstract

*Psilochasmus* Lühe, 1909 is a small genus of psilostomid digeneans parasitic in birds and characterized by the presence of a retractable tail-like structure at the posterior end of the body. Despite its low diversity, the taxonomic history of the genus is tumultuous, with opinions varying from recognizing only a single species to 11 nominal species. In this study, newly generated and previously available sequences of nuclear ribosomal DNA operon (ITS1+5.8S+ITS2; 28S) and partial NADH dehydrogenase (*nad*1) mtDNA gene sequences of *Psilochasmus* spp. from Europe (Palearctic), North America (Nearctic) and South America (Neotropics) were used to assess the diversity of *Psilochasmus* spp. and explore the phylogenetic interrelationships among members of genus and to distinguish between species. Based on combined morphological and molecular data, descriptions of two new *Psilochasmus* species from Europe and North America, *Psilochasmus slavaukrainii***sp. nov.** and *Psilochasmus urbeni***sp. nov.**, are provided, as well as the first morphological description of *P. oxyurus* specimens linked to DNA sequence data. In addition, a key to the identification of Psilochasmus spp. recognized as a result of this study has been constructed.

## ﻿Introduction

*Psilochasmus* Lühe, 1909 is a small genus of psilostomid digeneans (Echinostomatoidea Looss, 1902: Psilostomidae Looss, 1900) known to parasitize the intestines of their avian definitive hosts worldwide (Kostadinova 2005). The general morphology of *Psilochasmus* spp. is echinostome-like but lacking the cephalic collar typical of most echinostomatids. In addition, members of the genus are characterized by the presence of a protrusible, retractile, muscular tail-like process at the posterior end of the body (Kostadinova 2005).

The type-species of the genus, *Psilochasmus oxyurus* (Creplin, 1825), was originally described by Creplin (1825) from greater scaup *Aythya marila* (L.) (locality not provided). Creplin (1825) only provided a superficial description, but, later, Braun (1902) redescribed the species based on the material of Creplin (1825). Subsequently, ten other nominal *Psilochasmus* species were described; however, the validity of most of these species has been contested, which resulted in various synonymies (Skrjabin 1913; Travassos 1921; Ishii 1935; Yamaguti 1939, 1958, 1971; Gnedina 1946; Gupta 1957; Jaiswal 1957; Loos-Frank 1968; Oshmarin 1970; Jaiswal and Humayun 1972). In fact, some authors have considered the genus to be monotypic (Premvati 1969), whereas others have accepted various numbers of nominal species as valid (e.g., Stunkard and Dunihue 1931; Yamaguti 1939, 1958, 1971; Jaiswal and Humayun 1971; Fernandes et al. 2007).

In the present study, we generated partial sequences of nuclear ribosomal DNA operon (ITS1+5.8S+ITS2; 28S) as well as partial NADH dehydrogenase (*nad*1) mtDNA gene sequences of *Psilochasmus* spp. from Europe (Palearctic), North America (Nearctic) and South America (Neotropics). The newly generated 28S and *nad*1 sequences were used to explore the phylogenetic interrelationships among members of the genus and to distinguish between species. Through a combination of newly collected specimens, review of original descriptions and molecular phylogenies, we re-evaluate the constituent taxa of the genus. In addition, we provide descriptions of two new species of *Psilochasmus* and specimens of *P. oxyurus*.

## ﻿Materials and methods

### ﻿Sampling and morphological study

Adult digeneans were collected from the intestines of a variety of avian definitive hosts in Ukraine, USA, Argentina and New Zealand (Table 1). Live worms were removed from their hosts, briefly rinsed in saline, killed in hot water and fixed in 70% ethanol; dead worms were immediately fixed in 70% ethanol upon collection. Specimens for morphological study were stained with an aqueous alum carmine, dehydrated in an ethanol series of ascending concentrations, permanently mounted using dammar gum and studied with a DIC-equipped Olympus BX53 compound microscope (Tokyo, Japan) with a digital camera and drawing tube. All measurements are provided in micrometers. The measurements of the ventral sucker in the descriptions below include both the strongly muscular central part and the surrounding sub-tegumental rim. The type-series and voucher specimens are deposited in the collections of the Harold W. Manter Laboratory (HWML), University of Nebraska State Museum, Lincoln, Nebraska, USA. (Table 1), the University of Wisconsin – Stevens Point Parasitology Collection (UWSP – PARA) and the Museum of Southwestern Biology at the University of New Mexico, Albuquerque (MSB). In addition to our new material, we examined and identified some specimens of *Psilochasmus* from the collection of late Norman Dronen recently deposited in the MSB.

**Table 1.** Hosts, geographic origin, GenBank accession numbers and museum accession numbers of *Psilochasmus* spp. sequenced in this study. Museum abbreviations: Harold W. Manter Laboratory, HWML; Museum of Southwestern Biology, MSB; University of Wisconsin – Stevens Point Parasitology Collection (UWSP – PARA).

*Psilochasmus* spp.	Host species	Country	Museum No.	GenBank accession numbers	
				ITS region+28S	*nad*1
Psilochasmus cf. agilis	*Netta peposaca*	Argentina	–	PX111703*^†^	PX114582*
*Psilochasmus oxyurus*	*Anas crecca*	Ukraine	HWML-218106*	PX118872*	PX114583*
*Psilochasmus slavaukrainii* sp. nov.	*Anas clypeata*	Ukraine	HWML-218107*, 218108*	PX118873*	PX114584*
*P. slavaukrainii* sp. nov.	*Tadorna ferruginea*	Ukraine	HWML-218109*	PX118874*	PX114585*
*Psilochasmus urbeni* sp. nov.	*Aythya affinis*	USA	HWML-218111*, UWSP-PARA-8657–8659*, MSB:Para:24796	PX118875*^‡^	PX114586–PX114588*
*P. urbeni* sp. nov.	*Aythya marila*	USA	HWML-218110*	–	–
*P. urbeni* sp. nov.	*Melanitta americana*	USA	MSB:Para:35969	–	PX114589*
*Psilochasmus* sp.	*Mareca americana*	USA	MSB:Para:52035^§^	–	–
*Psilochasmus* sp.	*Aythya novaeseelandiae*	New Zealand	MSB:Para:20784	–	–

* New sequences and/or deposited slides; † 28S only; ‡ 5.8S+ITS2+28S only; § Specimens from the Norman Dronen collection.

### ﻿Molecular study

Genomic DNA was extracted from partial specimens according to the protocol of Tkach and Pawlowski (1999). Fragments of ribosomal and mitochondrial loci were amplified by polymerase chain reactions (PCR) using a T100 thermal cycler (Bio-Rad, California, USA). PCRs of 28S rRNA gene used the forward primer digL2 (5’−AAG CAT ATC ACT AAG CGG−3’) and reverse primer 1500R (5’−GCT ATC CTG AGG GAA ACT TCG−3’) (Tkach et al. 2003); the ITS region was amplified with forward primer ITS5 (5’–CGC CCG TCG CTA CTA CCG ATT G–3’) and reverse primer 300R (5’–CAA CTT TCC CTCACG GTA CTT G–3’) (Littlewood and Olson 2001; Snyder and Tkach 2007). The mitochondrial *nad*1 gene was amplified using the forward primer NDJ11 (5’−AGA TTC GTA AGG GGC CTA ATA−3’) and reverse primer NDJ2a (5’−CTT CAG CCT CAG CAT AAT−3’) (Morgan and Blair 1998; Kostadinova et al. 2003). PCRs were performed in a total volume of 25 μl using GoTaq G2 DNA Polymerase (Promega, Wisconsin, USA) according to the manufacturer’s protocol and an annealing temperature of 53 °C for rDNA and 45 °C for mtDNA reactions. Unfortunately, we were unable to amplify the ITS region of Psilochasmus cf. agilis and ITS1 of *Psilochasmus urbeni* sp. nov.; we were also unable to successfully amplify DNA from the extracted New Zealand specimen.

PCR products were purified using an ExoSAP-IT PCR clean-up enzymatic kit (Affymetrix, California, USA) and cycle sequenced with a MCLab BrightDye terminator chemistry (Molecular Cloning Laboratories, California, USA). PCR primers were used for sequencing reactions of 28S and *nad*1; PCR primers and internal primer d58F (5’−GCG GTG GAT CAC TCG GCT CGT G−3’) were used for sequencing the ITS region (Kudlai et al. 2015). BigDye sequencing clean up kit (Molecular Cloning Laboratories) was used to clean up sequencing reactions. Purified sequencing reactions were run on an ABI 3130 automated capillary sequencer (Thermo Fisher Scientific, Massachusetts, USA). Contiguous sequences were assembled using Sequencher v. 4.2 (GeneCodes Corp., Ann Arbor, Michigan, USA) and deposited in GenBank (Table 1).

The phylogenetic analyses were based on separate alignments of 28S and *nad*1. The ITS region was not used for phylogenetic analysis due to a lack of data from three species: P. cf. agilis, *P. urbeni* sp. nov. and *Psilochasmus* sp. of Koch (2004). The latter sequence clearly represents a *Psilochasmus* despite problems with its source. In GenBank this sequence is referred to as *Echinoparyphium* sp. while the figure in Koch (2004: fig. 3C) shows an echinostomatid that looks more similar to *Echinostoma* sp. Nevertheless, it is a sequence of a *Psilochasmus* sp. collected from snails in Australia. The alignment used to analyze 28S included four newly generated sequences and one previously published sequence of *Psilochasmus* spp. as well as 20 previously published sequences of other psilostomids; the *nad*1 alignment included 8 newly generated sequences of *Psilochasmus* spp. *Stephanoprora pseudoechinata* (Olsson, 1876) was selected as the outgroup of the 28S analysis based on the topology of Tkach et al. (2016); *Sphaeridiotrema pseudoglobulus* McLaughlin, Scott & Huffman, 1993 was used as the outgroup for the *nad*1 analysis based on the topology of our 28S analysis and availability of sequence. Sequences were aligned in MEGA7 using ClustalW (Kumar et al. 2016) and the alignments were trimmed to the length of the shortest sequences. The best fitting nucleotide substitution models were determined for both alignments using MEGA7 (Kumar et al. 2016). The general time-reversible model with estimates of invariant sites and gamma-distributed among-site variation (GTR+G+I) was used in the 28S analysis; Hasegawa-Kishino-Yano model with estimates of invariant sites and gamma-distributed among-site variation (HKY+G+I) was used in the *nad*1 analysis. The phylogenetic analyses were conducted using Bayesian Inference (BI) as implemented in MrBayes v. 3.2.6 software and Maximum Likelihood (ML) using MEGA7 (Ronquist and Huelsenbeck 2003; Kumar et al. 2016). The BI analyses were conducted as follows: Markov chain Monte Carlo (MCMC) chains were run for 3,000,000 generations, log-likelihood scores were plotted and only the final 75% of trees were used to produce the consensus trees. The number of generations was considered sufficient as the average standard deviation of split frequencies stabilized below 0.01. The branch supports in ML analyses were estimated based on 1,000 bootstrap pseudoreplicates. Pairwise nucleotide comparisons were performed using MEGA7.

## ﻿Results

### ﻿Molecular phylogenies

**Figure 1.**
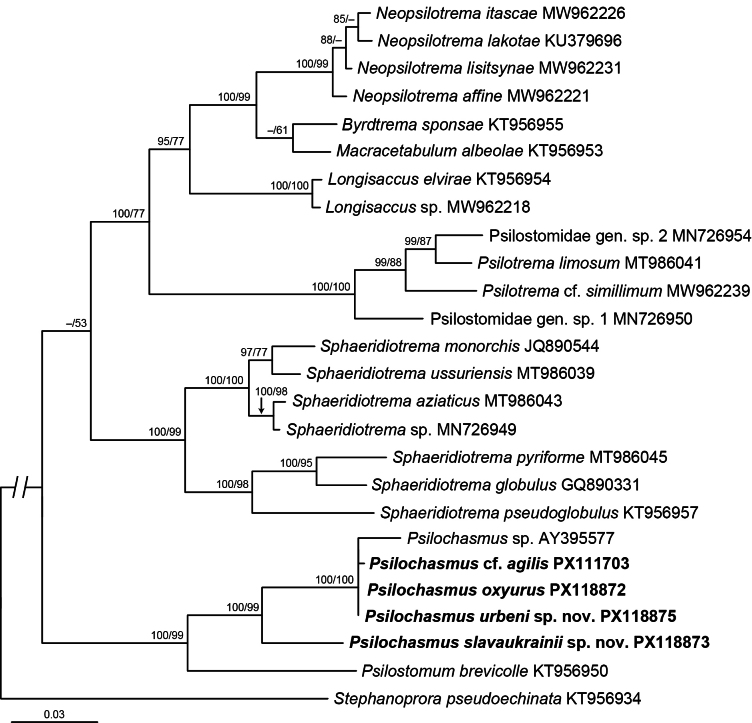
Phylogenetic interrelationships among the psilostomids based on Bayesian Inference (BI) and Maximum Likelihood (ML) analyses of partial 28S rRNA gene sequences. The topology of the BI analysis is presented with the BI posterior probability followed by the ML bootstrap values provided above the internodes. BI posterior probability values below 80% and ML bootstrap values below 50% are not shown. New sequences obtained in this study are in bold. The scale bar indicates the number of substitutions per site. GenBank accession numbers are provided after the names of all species.

After trimming to the length of the shortest sequence, the alignment used for the 28S analysis was 1,050 base pairs long; 6 sites were excluded due to ambiguous homology. The phylogeny based on 28S was well-resolved (Fig. 1) and similar to that of Achatz et al. (2021). A clade of *Psilochasmus* spp. + *Psilostomum brevicolle* (Creplin, 1829) (BI: 100% supported; ML: 99%) was positioned as a sister group to a weakly supported clade consisting of other psilostomids (BI: < 80% supported; ML: 53%). *Psilochasmus* spp. formed a strongly supported clade (BI: 100% supported; ML: 99%). *Psilochasmus slavaukrainii* sp. nov. was positioned as a sister group to the polytomic clade consisting of the remaining *Psilochasmus* spp. (BI: 100% supported; ML: 100%).

**Figure 2.**
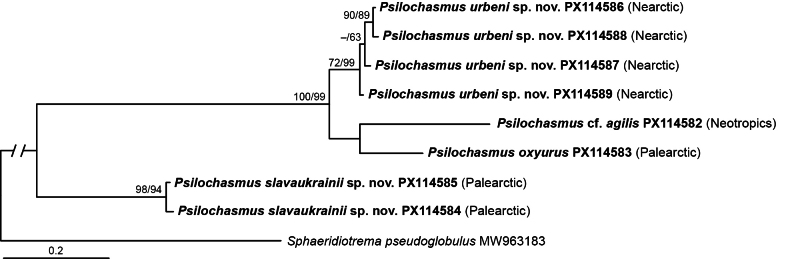
Phylogenetic interrelationships among *Psilochasmus* spp. based on Bayesian Inference (BI) and Maximum Likelihood (ML) analyses of partial *nad*1 mtDNA gene sequences. The topology of the BI analysis is presented with the posterior probability followed by the ML bootstrap values provided above the internodes. BI posterior probability values below 70% and ML bootstrap values below 50% are not shown. The new sequences obtained in this study are in bold. The scale bar indicates the number of substitutions per site. GenBank accession numbers and biogeographic realms are provided after the names of all species.

Upon trimming to the length of the shortest sequence, the alignment used for the *nad*1 analysis was 450 bases long; no sites were excluded. The resulting phylogeny (Fig. 2) was essentially identical to the topology of *Psilochasmus* spp. based on the 28S (Fig. 1). *Psilochasmus slavaukrainii* sp. nov. was positioned as a sister group to the clade (BI: 100% supported; ML: 99% supported) comprising the remaining *Psilochasmus* spp. (from the Palearctic, Nearctic and Neotropics). Both isolates of *P. slavaukrainii* sp. nov. formed a strongly supported clade (BI: 98% supported; ML: 94% supported). The clade that included *P. oxyurus* (from the Palearctic), P. cf. agilis (from the Neotropics) and *P. urbeni* sp. nov. (from the Nearctic) appeared as a polytomy. The four sequences of *P. urbeni* sp. nov. formed a subclade (BI: 72% supported; ML: 99% supported).

### ﻿Descriptions

Based on our review of literature and the morphology of existing nominal species of *Psilochasmus*, we only recognize *Psilochasmus agilis* Travassos, 1921, *Psilochasmus longicirratus* Skrjabin, 1913, *P. oxyurus*, *Psilochasmus skrjabini* Gnedina, 1946 and *Psilochasmus sphincteropharynx* Oshmarin, 1970 as valid species (see Discussion below).

### ﻿Taxonomy

Family Psilostomidae Looss, 1900

*Psilochasmus* Lühe, 1909

#### ﻿ Psilochasmus oxyurus

Taxon classificationAnimaliaPlagiorchiidaPsilostomidae

(Creplin, 1825)

062CC7ED-45FA-5BD5-8D7B-DEEB835A4C51

Fig. 3

##### Description.

Based on six adult specimens (measurements of illustrated specimen are given in text; measurements of entire series are given in Table 2). Body elongate, cylindrical, somewhat wider near level of testes, 4,189 × 615, with narrow muscular, retractile tail-like process. Body length to width ratio 6.8. Ratio of body width at level of testes to body width at level of ventral sucker 1.0. Tegument armed. Forebody length represents 26% of body length. Oral sucker subterminal, elongate-oval, 294 × 282, sometimes withdrawn under surface of tegument. Ventral sucker protuberant with deep cavity, consisting of strongly muscular portion and extensive surrounding sub-tegumental rim, larger than oral sucker, 609 × 586. Oral sucker to ventral sucker width ratio 0.5. Prepharynx short, not observed in holotype. Pharynx muscular, elongate-oval, smaller than oral sucker, 145 × 127. Oral sucker to pharynx length ratio 2.0; oral sucker to pharynx width ratio 2.2. Esophagus muscular, bifurcating anterior to level of ventral sucker, 328. Ceca thin-walled, extending to near posterior end of body.

**Table 2.** Morphometric characters of the *Psilochasmus* spp. described in the present study: ranges followed by mean in parentheses.

Species	*P. oxyurus*	*P. slavaukrainii* sp. nov.	*P. urbeni* sp. nov.
**Host**	*Anas crecca*	*Anas clypeata*	*Aythya marila*, *Aythya affinis*
**Locality**	Ukraine	Ukraine	USA
**Number of specimens (*n*)**	6	4	5
Body length	2,415–4,189 (3,047)	2,076–3,219 (2,623)	6,000–7,020 (6,674)
Body width at level of testes	449–615 (534)	660–759 (699)	661–1,405 (1,080)
Body length to width ratio	5.4–6.8 (5.9)	3.1–4.3 (3.7)	5–7.4 (5.8)
Body width ratio at levels of testes:ventral sucker	1–1.3 (1.1)	1.5–1.8 (1.7)	0.9–1.5 (1.2)
Forebody length as (% of body length)	26–37% (32%)	30–36% (34%)	27–33% (30%)
Oral sucker length	213–294 (241)	192–259 (222)	318–455 (387)
Oral sucker width	209–282 (231)	195–206 (201)	284–417 (370)
Ventral sucker length	375–609 (460)	312–389 (343)	402–785 (632)
Ventral sucker width	387–586 (452)	330–426 (374)	520–830 (698)
Oral sucker to ventral sucker width ratio	0.5–0.6 (0.5)	0.5–0.6 (0.5)	0.5–0.6 (0.5)
Prepharynx	0–31 (11)	0 (0)	0–48 (19)
Pharynx length	107–145 (120)	103–143 (127)	224–301 (281)
Pharynx width	74–128 (106)	73–100 (90)	194–376 (282)
Oral sucker to pharynx length ratio	1.8–2.3 (2)	1.4–1.9 (1.7)	1.1–1.5 (1.4)
Oral sucker to pharynx width ratio	1.6–2.8 (2.3)	2.1–2.7 (2.3)	1.1–1.5 (1.3)
Esophagus length	328–456 (370)	182–482 (330)	537–923 (673)
Anterior testis length	286–485 (355)	217–269 (237)	327–810 (646)
Anterior testis width	174–258 (223)	222–238 (230)	248–617 (468)
Posterior testis length	308–517 (386)	221–345 (279)	612–951 (820)
Posterior testis width	148–236 (191)	157–208 (189)	234–528 (395)
Cirrus-sac length	487–1197 (769)	421–501 (462)	1,690*
Cirrus-sac width	53–100 (72)	68–80 (75)	226*
Ovary length	110–180 (133)	95–128 (115)	108–273 (225)
Ovary width	103–148 (118)	82–128 (113)	108–278 (228)
Number of eggs	2–17 (6)	1–5 (4)	1–137 (78)
Egg length	79–99 (88)	84–101 (94)	87–113 (99)
Egg width	49–60 (54)	50–66 (60)	55–75 (66)

* Only observed in holotype.

**Figure 3.**
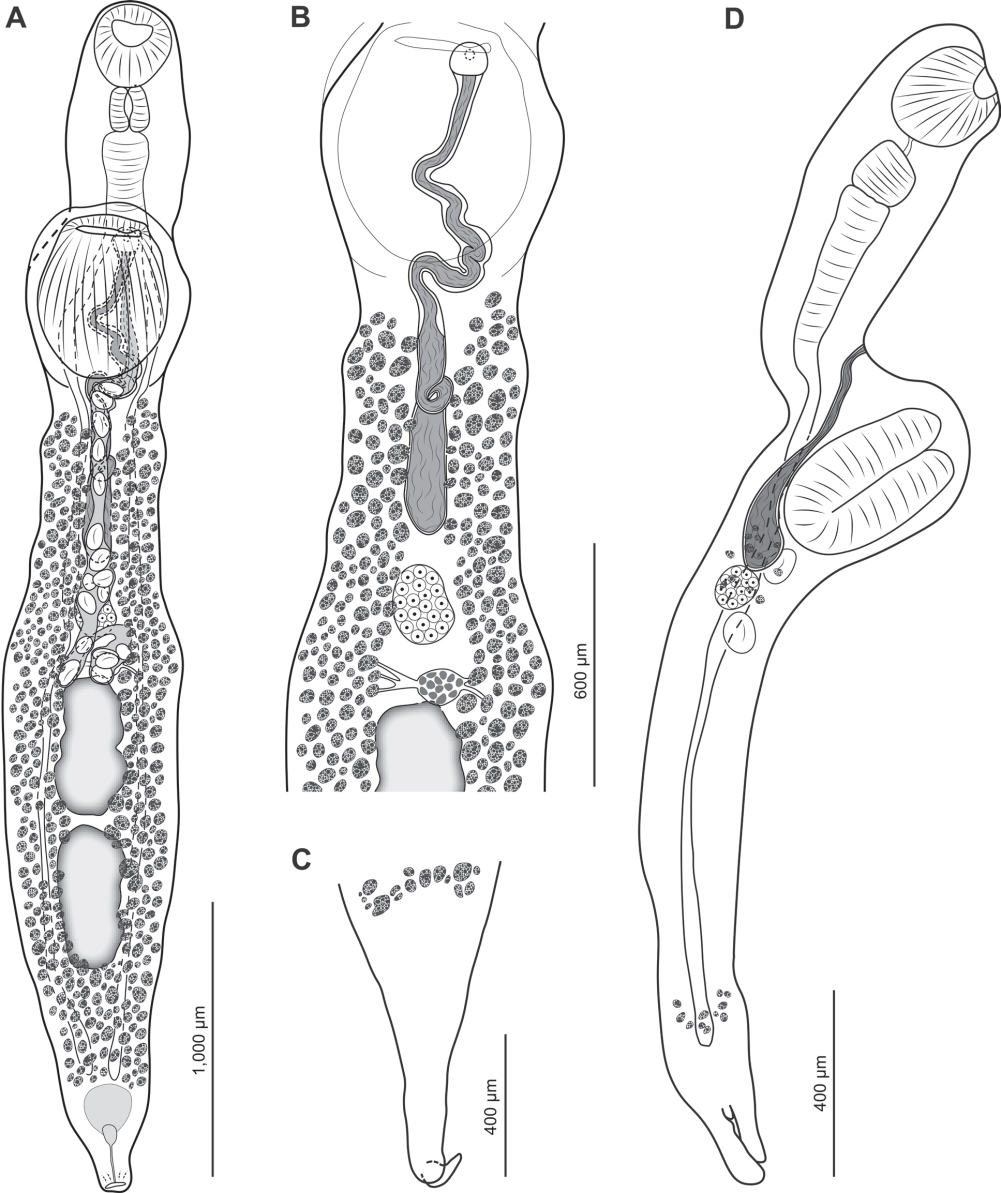
*Psilochasmus oxyurus* vouchers. A. Entire, ventral view; B. Middle of body, ventral view; uterus and eggs omitted; C. Posterior end with tail protruding, ventral view; D. Entire, lateral view.

Testes tandem, in posterior half of body, weakly or strongly lobate. Anterior testis 485 × 258; posterior testis 517 × 236. Cirrus-sac elongate, slender, reaching level of ovary or anterior to it, 1,197 × 100. Internal seminal vesicle unipartite, tubular, with broader proximal part. Pars prostatica indistinct. Genital pore immediately anterior to level of ventral sucker.

Ovary oval, median or submedian, pretesticular, 180 × 148. Mehlis’ gland between level of ovary and anterior testis. Uterine seminal receptacle present. Laurer’s canal not observed. Vitellarium distributed throughout most of hind body length, absent in tail; most follicles lateral to gonads, uterus and cirrus-sac. Vitelline reservoir between level of ovary and anterior testis. Eggs not numerous, ≤17 present, 79–99 × 49–60.

Excretory pore subterminal. Excretory bladder not readily observed.

##### Taxonomic summary.

**Type host**: *Aythya marila* (L.) (Anseriformes: Anatidae).

**Host in this study**: *Anas crecca* L. (Anseriformes: Anatidae).

**Site of infection**: small intestine.

**Locality in this study**: Skadovsk District, Kherson Region, Ukraine (46°07'55.6"N, 32°13'40.7"E).

**Specimens deposited**: Vouchers: HWML-218106, labeled *Anas crecca*, small intestine, Skadovsk District, Kherson Region, Ukraine, 31 Aug 2011, coll. V. V. Tkach.

**Representative DNA sequences**: PX118872 (ITS region + 28S); PX114583 (*nad*1).

##### Remarks.

The original description of the species by Creplin (1825) and redescription by Braun (1902) lack most details and measurements provided in descriptions of other echinostomatoid taxa. Despite this, the morphology (quantitative and qualitative characters) of our specimens of *P. oxyurus* closely conforms to the original description by Creplin (1825) and redescription by Braun (1902) (Table 2). Importantly, we provide molecular data associated with our described specimens of *P. oxyurus*.

#### ﻿ Psilochasmus slavaukrainii

Taxon classificationAnimaliaPlagiorchiidaPsilostomidae

Achatz, Morton, Orlofske & Tkach sp. nov.

C27DBD6E-7508-5C65-AB76-58AEF60E9353

https://zoobank.org/1A7B0DC3-DDA7-438A-902B-E2F3EB55D90E

Fig. 4a, b

##### Type material.

***Holotype***: HWML-218107, labeled *Anas clypeata*, small intestine, Skadovsk District, Kherson Region, Ukraine, 9 Nov 2002, coll. V. V. Tkach. ***Paratypes*** (3 slides), labeled identical to holotype: HWML-218108. Vouchers (juveniles; 1 slide): HWML-218109, labeled *Tadorna ferruginea*, small intestine, Skadovsk District, Kherson Region, Ukraine, 30 Oct 2002, coll. V.V. Tkach.

**Figure 4.**
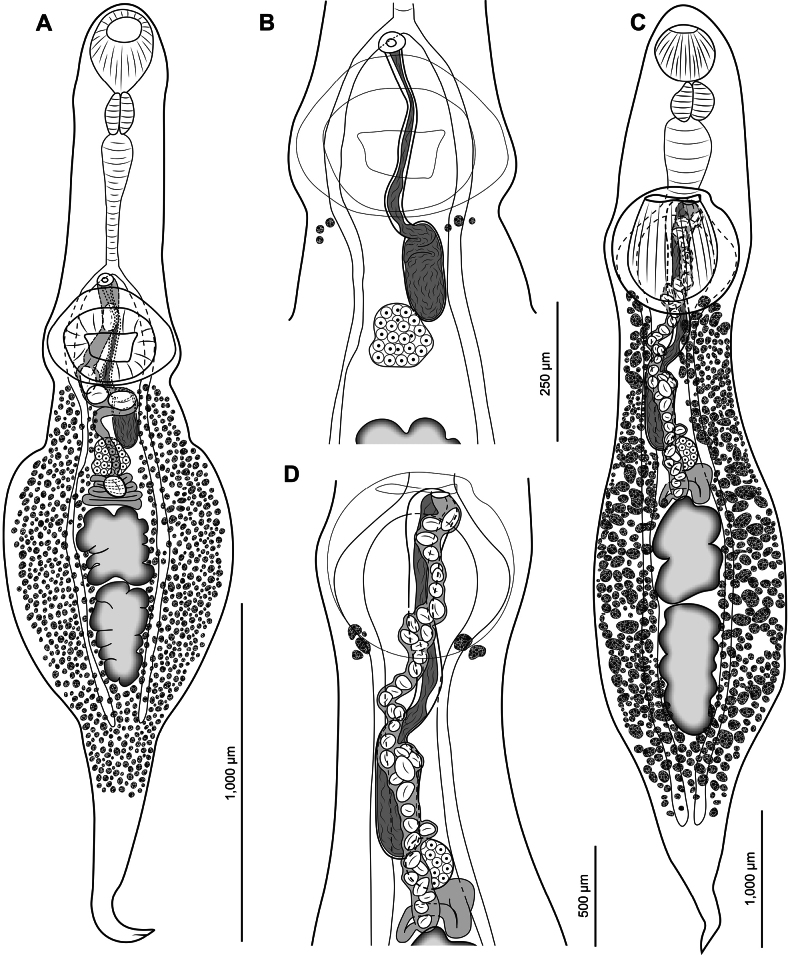
New *Psilochasmus* spp. A. *Psilochasmus slavaukrainii* sp. nov. holotype, entire, ventral view; B. *P. slavaukrainii* sp. nov. holotype, middle of body, ventral view; uterus and eggs omitted; C. *Psilochasmus urbeni* sp. nov. holotype, entire, ventral view; D. *P. urbeni* sp. nov. holotype, middle of body, ventral view; uterus and eggs omitted.

##### Description.

Based on four adult specimens (measurements of holotype are given in text; measurements of entire series are given in Table 2). Body elongate, 3,219 × 759, widest near level of testes; forebody cylindrical; hindbody with strong lateral expansion with narrow muscular, retractable tail-like process,. Body length to width ratio 4.2. Ratio of body width at level of testes to body width at level of ventral sucker 1.7. Tegument armed. Forebody length represents 35% of body length. Oral sucker subterminal, oval, 259 × 206. Ventral sucker protuberant with deep cavity, consisting of strongly muscular portion and extensive surrounding sub-tegumental rim, 312 × 426. Oral sucker to ventral sucker width ratio 0.5. Prepharynx not observed. Pharynx muscular, elongate-oval, 143 × 100. Oral sucker to pharynx length ratio 1.8; oral sucker to pharynx width ratio 2.1. Esophagus muscular, bifurcating anterior to level of ventral sucker, 482. Ceca thin-walled, extending posterior to level of posterior testis.

Testes tandem, in posterior half of body, lobate. Anterior testis 269 × 238; posterior testis 345 × 199. Cirrus-sac elongate, slender, reaching level of ovary or anterior to it, 421 × 80. Internal seminal vesicle unipartite, tubular, with broader proximal part. Pars prostatica indistinct. Genital pore immediately anterior to anterior margin of ventral sucker.

Ovary oval, median or submedian, pretesticular, posterior to level of ventral sucker, 128 × 128. Mehlis’ gland between level of ovary and anterior testis. Uterine seminal receptacle present. Laurer’s canal not observed. Vitellarium distributed throughout most of hindbody length, absent in tail; most follicles lateral to gonads, uterus and cirrus-sac. Vitelline reservoir between level of ovary and anterior testis (not readily observed in holotype). Eggs few, 5 in holotype, 84–101 × 50–66.

Excretory pore positioned near tip of tail. Excretory bladder not readily observed.

##### Taxonomic summary.

**Type host**: *Anas clypeata* (L.) (Anseriformes: Anatidae).

**Other host (only juveniles collected)**: *Tadorna ferruginea* (Pallas) (Anseriformes: Anatidae).

**Site**: small intestine.

**Locality**: Skadovsk District, Kherson Region, Ukraine; 46°26'38.3"N, 32°01'44.9"E.

**Representative DNA sequences**: PX118873 (ITS region + 28S); PX114584 (*nad*1).

##### Etymology.

This species is named in honor of the national salute in the country in which it was collected.

##### Diagnosis.

These digeneans clearly belong to *Psilochasmus* based on the echinostome-like body that lacks a cephalic collar and the presence of a protrusible, retractile, muscular tail-like process at the posterior end of the body. The body of *P. slavaukrainii* sp. nov. has a strong lateral expansion immediately posterior to the level of the ventral sucker that is absent in properly relaxed mature congeners. It is noteworthy that the original description of *P. agilis* by Travassos (1921) did not mention such a lateral expansion. However, Szidat (1957) illustrated ‘younger’ *P. agilis* specimens (referred to as *P. oxyurus*) that exhibited this trait. At the same time, the mature adult stage illustrated by Szidat (1957) had only a slight widening of the body.

*Psilochasmus slavaukrainii* sp. nov. is a much smaller digenean compared with *P. agilis*. For instance, the body length of mature specimens is only 2,076–3,219, whereas the body length of mature *P. agilis* exceeds 4,000. The forebody of the new species represents 30–36% of the body length, whereas in *P. agilis* it is approximately 25%. The new species is distributed in the Palearctic, whereas *P. agilis* is restricted to the Neotropics, and possibly Nearctic (see Discussion below). *Psilochasmus slavaukrainii* sp. nov. and P. cf. agilis differ by 2.1% in the partial 28S sequences and 16.8–17.2% in the partial *nad*1 sequences (Table 3).

**Table 3.** Divergence percentages among *Psilochasmus* spp. resulting from pairwise sequence comparisons of 458 base pair long alignment of the partial *nad*1 gene (above diagonal), 1,141 base pair long alignment of the partial 28S gene (below diagonal, before slash) and 1,257 base pair long alignment of 5.8S+ITS2 (below diagonal, after slash). GenBank numbers for the *nad*1 sequences are provided in the top row. GenBank numbers for ribosomal sequences are provided in the first column.

		1.	2.	3.	4.	5.	6.	7.	8.	9.
		PX114582	PX114583	PX114585	PX114584	PX114586	PX114587	PX114588	PX114589	–
**1.**	P. cf. agilis PX111703	–	9.8	16.8	17.2	9.6	9.4	9.8	9.0	–
**2.**	*P. oxyurus*PX118872*	0.1/–	–	16.4	16.8	6.3	6.1	6.6	5.7	–
**3.**	*P. slavaukrainii* sp. nov. PX118874	2.1/–	2.0/11.5	–	0.4	15.5	15.7	15.7	15.3	–
**4.**	*P. slavaukrainii* sp. nov. PX118873	2.1/–	2.0/11.5	0/0	–	15.9	16.2	16.2	15.7	–
**5.**	*P. urbeni* sp. nov. PX118875	0.1/–	0/0.2	2.0/11.5	2.0/11.5	–	0.7	0.2	0.7	–
**6.**	*P. urbeni* sp. nov. –	–/–	–/–	–/–	–/–	–/–	–	0.9	0.4	–
**7.**	*P. urbeni* sp. nov. –	–/–	–/–	–/–	–/–	–/–	–/–	–	0.9	–
**8.**	*P. urbeni* sp. nov. –	–/–	–/–	–/–	–/–	–/–	–/–		–	–
**9.**	*Psilochasmus* sp. AY395577	0.5/–	0.4/–	2.5/–	2.5/–	0.4/–	–/–	–/–	–/–	–

*28S region from this specimen is identical to the 28S sequence of *P. oxyurus* collected from *Anas platyrhynchos* in Kherson region, Ukraine available in GenBank (AF151940).

The oral sucker to pharynx width ratio of *P. slavaukrainii* sp. nov. (2.1–2.7) is greater than in *P. longicirratus* (1.6 based on the original drawing). The new species is shorter (2,076–3,219) compared to *P. longicirratus* (3,740–5,000). Furthermore, the suckers and pharynx of *P slavaukrainii* sp. nov. are much smaller (oral sucker 192–259 × 195–206; ventral sucker 312–389 × 330–426; pharynx 103–143 × 73–100) compared to *P. longicirratus* (oral sucker 340 in diameter; ventral sucker 640 in diameter; pharynx 255 × 204). However, the testes of the new species are typically larger (anterior testis 217–269 × 222–238; posterior testis 221–345 × 157–208) compared to *P. longicirratus* (testes 170 in diameter). The eggs of the new species (84–101) are much smaller than those of *P. longicirratus* (116–124).

*Psilochasmus slavaukrainii* sp. nov. and *P. oxyurus* are very similar morphologically. The oral sucker to pharynx length ratio is generally smaller in the new species (1.4–1.9, mean 1.8) compared to *P. oxyurus* (1.8–2.3, mean 2.0 in our material). The post-testicular field is longer in the new species (27–30%, mean 28% of body length) compared to *P. oxyurus* (23% of body length based on the illustration of Braun (1902) (14–25%, mean 19% of body length in present material). Despite being similar morphologically, these species differ by 2.0% in the 28S, 11.5% in the 5.8S+ITS2, and 16.4–16.8% in the partial *nad*1 sequences (Table 3).

*Psilochasmus slavaukrainii* sp. nov. has a well-developed esophagus (182–482 long), whereas the ceca of *P. skrjabini* bifurcate almost immediately posterior to the pharynx. The new species is much smaller in body length (2,076–3,219 in the new species vs 6,750 in *P. skrjabini*), oral sucker width (195–206 in *P. slavaukrainii* sp. nov. vs 400 in *P. skrjabini*), ventral sucker size (312–389 × 330–426 in *P. slavaukrainii* sp. nov. vs 780 × 600 in *P. skrjabini*) and pharynx size (103–143 × 73–100 in *P. slavaukrainii* sp. nov. vs 200 × 250 in *P. skrjabini*).

*Psilochasmus slavaukrainii* sp. nov. lacks a distinct additional muscular sphincter at the anterior end of the pharynx, whereas such a structure is present in *P. sphincteropharynx*. The pharynx of *P. slavaukrainii* sp. nov. (103–143 × 73–100) is also smaller than *P. sphincteropharynx* (150–162 × 160–170). *Psilochasmus slavaukrainii* sp. nov. has a smaller body (2,076–3,219) than *P. sphincteropharynx* (4,200–4,250). The oral sucker of these species is similar in size, or slightly smaller in the new species, whereas the ventral sucker is noticeably larger in the new species (312–389 × 330–426) compared to *P. sphincteropharynx* (270 in diameter). Eggs are somewhat shorter in *P. slavaukrainii* sp. nov. (84–101) compared to those of *P. sphincteropharynx* (105–110).

It is worth noting that the specimen of *P. oxyurus* illustrated by Bykhovskaja-Pavlovskaja (1962: fig. 68), from the mallard *Anas platyrhynchos* L. (collected from an unknown locality in the former Soviet Union) appears to be essentially identical to *P. slavaukrainii* sp. nov.

No variation was detected in ribosomal loci (ITS1+5.8S+ITS2+28S) between the two isolates of the new species, whereas a 0.4% variation was observed between two isolates in the partial *nad*1 sequences (Table 3).

#### ﻿ Psilochasmus urbeni

Taxon classificationAnimaliaPlagiorchiidaPsilostomidae

Achatz, Morton, Orlofske & Tkach sp. nov.

D2E7F4B5-1BC7-5700-95BC-B9F15BDFA619

https://zoobank.org/526DBBCA-2017-4C43-A371-53F44D21C0BE

Fig. 4c, d

##### Type material.

***Holotype***: HWML-218110, labeled *Aythya marila*, small intestine, Stump Lake, Nelson County, North Dakota, USA, 6 Nov 2006, coll. V.V. Tkach. ***Paratypes***: HWML-218111 (hologenophore), *Aythya affinis*, small intestine, Lake Winnibigoshish, Itasca County, Minnesota, USA, 1 Nov 2007, coll. V.V. Tkach; UWSP – PARA (3 paratypes): *Aythya affinis*, small intestine, Green Bay, Lake Michigan, Oconto County, Wisconsin, USA, 24 Nov 2019, coll. S.A. Orlofske.

##### Description.

Based on 5 specimens (measurements of holotype are given in text; measurements of entire series are given in Table 2). Body elongate, cylindrical, widest near level of testes, 6,711 × 1,307, with narrow, retractable, muscular tail-like process. Body length to width ratio 5.1. Ratio of body width at level of testes to body width at level of ventral sucker 1.3. Tegument armed. Forebody length represents 35% of body length. Oral sucker subterminal, subspherical, 422 × 417. Ventral sucker protuberant with deep cavity, consisting of strongly muscular portion and extensive surrounding sub-tegumental rim, 785 × 830. Oral sucker to ventral sucker width ratio 0.5. Prepharynx not observed. Pharynx muscular, subspherical, 287 × 290. Oral sucker to pharynx length ratio 1.5; oral sucker to pharynx width ratio 1.4. Esophagus muscular, bifurcating immediately anterior to level of ventral sucker, 675. Ceca thin-walled, extending to near posterior end of body.

Testes tandem, in posterior half of body, lobulated. Anterior testis 705 × 436; posterior testis 901 × 408. Cirrus-sac elongate, slender, reaching level of ovary or anterior to it, 1,690 × 226. Internal seminal vesicle unipartite, tubular, with broader proximal part. Pars prostatica indistinct. Genital pore immediately anterior to level of ventral sucker.

Ovary subspherical, median or submedian, pretesticular, posterior to level of ventral sucker, 232 × 238. Mehlis’ gland between level of ovary and anterior testis. Uterine seminal receptacle present. Laurer’s canal not observed. Vitellarium distributed throughout most of hind body length, absent in tail, with most follicles lateral to gonads, uterus and cirrus-sac, confluent posterior to testes. Vitelline reservoir (not readily observed in holotype) between level of ovary and anterior testis. Eggs numerous, 42 in holotype, ≤137 in paratype, 90–104 × 62–70.

Excretory pore not observed. Excretory bladder not readily observed.

##### Taxonomic summary.

**Type host**: *Aythya marila* (L.) (Anseriformes: Anatidae).

**Other hosts**: *Aythya affinis*, *Melanitta americana* (Swainson).

**Site**: small intestine.

**Type locality**: Stump Lake, Nelson County, North Dakota, USA; 47°53'15.5"N, 98°18'11.3"W.

**Other localities**: Lake Winnibigoshish, Itasca County, Minnesota, U.S.A.; 47°27'14.8"N, 94°16'30.9"W; Green Bay, Lake Michigan, Oconto County, Wisconsin, USA; 44°48'51.2"N, 87°52'54.7"W; Monroe County, Florida.

**Specimens deposited**: The type series consists of 5 fully mature specimens (one is a hologenophore).

**Representative DNA sequences**: PX118875 (5.8S+ITS2+28S), PX114586 (*nad*1).

##### Etymology.

This species is named for Bruce Urben (Wisconsin Waterfowl Association) and his family for their leadership in waterfowl conservation, support of wetland habitat restoration, and donation of numerous birds for parasitology research.

##### Diagnosis.

*Psilochasmus urbeni* sp. nov. belongs to the genus based on its echinostome-like body that lacks a cephalic collar and the presence of a protrusible, retractile, muscular tail-like process at the posterior end of the body. This new species has an oral sucker that is only slightly wider than the pharynx (oral sucker to pharynx width ratio 1.1–1.5, mean 1.3), whereas that of other *Psilochasmus* spp. is typically much wider than the pharynx (oral sucker to pharynx width ratio 1.6–2.8 based on the present study, original descriptions and illustrations).

The body length of *P. urbeni* sp. nov. (6,000–7,020) is greater than *P. agilis* (4,500), although the suckers and ovary in the two species are similar in size. The esophagus of *P. urbeni* sp. nov. (537–923) is much longer compared to *P. agilis* (270 based on the original illustration). *Psilochasmus urbeni* sp. nov. and P. cf. agilis differ by 0.1% in the 28S and 9.4–9.8% in the partial *nad*1 sequences (Table 3).

*Psilochasmus urbeni* sp. nov. is much longer than *P. longicirratus* (body length 6,000–7,020 in the new species vs 3,740–5,000 in *P. longicirratus*). Despite the difference in body length, all structures and organs overlap in size, except for testes; the testes of the new species are much larger (anterior testis 327–810 × 248–617; posterior testis 612–951 × 234–528) compared to *P. longicirratus* (testes 170 in diameter). The eggs of *P. urbeni* sp. nov. (87–113, mean 99) are generally smaller than in *P. longicirratus* (116–124).

Similar to the previous comparisons, the body length of *P. urbeni* sp. nov. (6,000–7,020) is greater than in *P. oxyurus* (2,415–4,189 in new material). The esophagus of *P. urbeni* sp. nov. (537–923) is much longer than in *P. oxyurus* (328–456 in new material). Both suckers and the pharynx are larger in the new species compared to *P. oxyurus* (Table 2). The oral sucker to pharynx length ratio is noticeably smaller in *P. urbeni* sp. nov. (1.1–1.5, mean 1.4) than in *P. oxyurus* (1.8–2.3, mean 2.0 in new material). These species differ by 0% in the 28S, 0.2% in the 5.8S+ITS2, and 6.3% in the partial *nad*1 sequences (Table 3).

*Psilochasmus urbeni* sp. nov. has a well-developed esophagus (537–923 long), whereas the cecal bifurcation of *P. skrjabini* is situated essentially immediately posterior to the pharynx. The oral sucker of *P. urbeni* sp. nov. (318–455) is also much longer than that of *P. skrjabini* (200).

The body of *P. urbeni* sp. nov. lacks the strong lateral expansion immediately posterior to the level of the ventral sucker that is present in *P. slavaukrainii* sp. nov. *Psilochasmus urbeni* sp. nov. is also a much larger digenean compared to *P. slavaukrainii* sp. nov. in most regards, including body, sucker, and pharynx sizes as well as esophageal length (Table 2). For example, the body length of *P. urbeni* sp. nov. is more than twice that of *P. slavaukrainii* sp. nov. (6,000–7,020 vs 2,076–3,219). These species differ by 2% in the 28S, in 11.5% in the 5.8S+ITS2, and 15.3–16.2% in the partial *nad*1 sequences (Table 3).

*Psilochasmus urbeni* sp. nov. lacks a distinct additional muscular sphincter at the anterior end of pharynx, which is present in *P. sphincteropharynx*. Otherwise, *P. urbeni* sp. nov. is a larger species as compared with *P. sphincteropharynx*. The body length of *P. urbeni* sp. nov. (6,000–7,020) is noticeably greater than that of *P. sphincteropharynx* (4,200–4,250). Both suckers and the pharynx are larger in the new species (oral sucker 318–455 × 284–417; ventral sucker 402–785 × 520–830; pharynx 224–301 × 194–376) compared with *P. sphincteropharynx* (oral sucker 240–270 × 220–230; ventral sucker 270 in diameter; pharynx 150–162 × 160–170).

Intraspecific variation of 0.2–0.9% was detected among *nad*1 sequences of *P. urbeni* sp. nov. (Table 3).

## ﻿Discussion

In the present study, we have provided descriptions of two new species, *P. slavaukrainii* sp. nov. (from Europe) and *P. urbeni* sp. nov. (from North America) and have also provided the first description of *P. oxyurus* associated with DNA sequence data. In the past, some authors have considered *P. oxyurus* to be the sole member of the genus (Premvati 1969), whereas others have recognized several species (Skrjabin 1913; Travassos 1921; Ishii 1935; Yamaguti 1939; Gnedina 1946; Gupta 1957; Jaiswal 1957; Loos-Frank 1968; Oshmarin 1970; Jaiswal and Humayun 1972). Our study has clearly demonstrated that the genus comprises at least 5 species based on genetic data.

Both phylogenetic analyses (Figs 1, 2) resulted in trees with similar topologies. Unfortunately, with the current dataset we were unable to reconstruct the sequence of dispersal events between biogeographic realms.

### ﻿Genetic variation

The genetic distances variation among *Psilochasmus* spp. varied significantly between different loci. Only 0–2.5% variation was detected among the partial 28S sequences across the genus (Table 3). In contrast, a much greater 0.2–11.5% and 5.7–17.2% interspecific variation was detected among the 5.8S+ITS2 and *nad*1 sequences, respectively. *Psilochasmus slavaukrainii* sp. nov. was the most divergent in these loci among all species included in our analyses. It differed from other congeners by 11.5% and 15.3–17.2% in sequences of the 5.8S+ITS2 and *nad*1, respectively, whereas all other members of the genus differ by 0.2% in the 5.8S+ITS2 sequences (only one comparison currently possible) and 5.7–9.8% difference in the *nad*1 sequences (Table 3). No intraspecific variation was detected among the 28S and ITS region sequences, whereas ≤ 0.9% intraspecific variation was detected among the *nad*1 sequences. Based on the present data, *nad*1 provides better resolution for differentiation between *Psilochasmus* spp.

### ﻿The validity of *Psilochasmus agilis*

Specimens identified as *P. oxyurus* (originally described from Europe) have been reported and described from a variety of avian hosts in North and South America. However, the identities of some of these digeneans have been the subject of debate. *Psilochasmus agilis* was described by Travassos (1921, 1929) based on specimens from the white-cheeked pintail *Anas bahamensis* L. collected in Brazil. Gupta (1957) synonymized *P. agilis* with *P. oxyurus*, but Yamaguti (1971) did not recognize this synonymy. Szidat (1957) described the life cycle of “*P. oxyurus*” based on stages from naturally infected *Heleobia australis* (d’Orbigny) (referred to as *Littoridina australis*) in Argentina. Adults described in that study were only obtained from laboratory infections of young ‘ducks and chickens’. Fernandes et al. (2007) provided a description of *P. oxyurus* specimens from a naturally infected graylag goose *Anser anser* (L.) in Brazil. These authors did not dispute the synonymy of *P. agilis* with *P. oxyurus*. We have generated DNA sequences from South American (Argentinean) specimens which we designate as Psilochasmus cf. agilis. While these specimens likely represent *P. agilis*, the poor quality of our specimens precludes detailed morphological comparisons. Psilochasmus cf. agilis exhibits 0.1–2.1% and 9.0–17.2% divergence from congeners, including *P. oxyurus*, in partial sequences of the 28S and *nad*1, respectively (Table 3). Based on molecular comparison, we agree with Yamaguti (1971) and reject the synonymy of *P. agilis* with *P. oxyurus*. Unfortunately, there are no apparent morphological differences in adults that can be used to reliably separate *P. oxyurus* and *P. agilis*. At present, molecular comparisons and geographic distribution (Palearctic vs Neotropics) are the best characteristics to distinguish these species.

### ﻿*Psilochasmus oxyurus* in North America

Stunkard and Dunihue (1931) described specimens identified as *P. oxyurus* from a “duck” in New York, USA, while Premvati (1969) described specimens from a mallard and greater scaup in Florida, USA. However, these North American specimens exhibit several morphological differences from *P. oxyurus* in Europe. For instance, the ratio of the body width at the level of the testes to that at the level of the ventral sucker is 1.0–1.3 in *P. oxyurus* (based on the present study) and 1.4 in *P. agilis* (based on the original illustration), whereas this ratio is 1.5 based on the illustrations of Stunkard and Dunihue (1931) and Premvati (1969). The oral sucker to pharynx length ratio is greater in *P. oxyurus* from Europe (1.7–2.4 based on the present study) and *P. agilis* from South America (1.7) compared with North American specimens reported as *P. oxyurus* (1.5–1.7 based on the illustrations of the above authors). Body length in the specimens of Stunkard and Dunihue (1931) and Premvati (1969) (4,200–6,900) is greater than in both *P. oxyurus* from Europe collected in the present study (2,415–4,189) and *P. agilis* (4,500). The eggs of *P. oxyurus* (79–100 long; Braun 1902; present study) and *P. agilis* (99) are somewhat smaller compared with those of the specimens of Stunkard and Dunihue (1931) (100–120 long) and Premvati (1969) (90–120 long).

Likewise, these specimens are not entirely consistent with *P. urbeni* sp. nov., also from North America. The body length of the specimens of Stunkard and Dunihue (1931) and Premvati (1969) (4,200–6,900) is similar to that of *P. urbeni* sp. nov. (6,000–7,020). However, the body length to width ratio of *P. urbeni* sp. nov. (5.0–7.4, mean 5.8) is greater than in the specimens of both Stunkard and Dunihue (1931: fig. 2) and Premvati (1969: fig. 1, 2) based on the original illustrations (3.5–4.3). At the same time, the ratio of the body width at the level of the testes to that at the level of the ventral sucker in *P. urbeni* sp. nov. is lower (0.9–1.5, mean 1.2) than that in the specimens of Stunkard and Dunihue (1931) and Premvati (1969) (1.3–1.5). More apparently, the oral sucker to pharynx length and width ratios (1.1–1.5 and 1.1–1.5, respectively) are smaller in *P. urbeni* sp. nov. compared with the specimens of Stunkard and Dunihue (1931) and Premvati (1969) based on the original illustrations (oral sucker to pharynx length ratio 1.6–1.7; oral sucker:pharynx width ratio 1.8–1.9).

Similarly, both specimens of the *Psilochasmus* sp. collected by Dronen from *Mareca americana* in Texas and deposited in the MSB demonstrate some features consistent with both *P. oxyurus* and *P. urbeni* sp. nov. The body length of Dronen’s specimens (5,725–6,725) is greater than that of *P. oxyurus* from the present study (2,415–4,189) and *P. agilis* (4,500), but similar to that of *P. urbeni* sp. nov. (6,000–7,020). At the same time, the oral sucker to pharynx length and width ratios of Dronen’s material (2–2.9 and 2.4–2.8, respectively) are similar to those of *P. oxyurus* (1.8–2.3 and 1.6–2.8, respectively) and *P. agilis* (1.7 and 2.2, respectively) but are much greater than in *P. urbeni* sp. nov. (1.6–1.7 and 1.8–1.9, respectively). However, without DNA sequences, we cannot confidently assign species names to the specimens of Stunkard and Dunihue (1931), Premvati (1969), and Dronen. It is possible that these materials represent additional morphological variation of *P. urbeni* sp. nov. Alternatively, they may represent *P. agilis* in the southern region of the Nearctic. Additional collections of *Psilochasmus* from the southernmost parts of the USA are needed to confirm the identification of these digeneans.

### ﻿Old World *Psilochasmus* species

It is likely that at least five *Psilochasmus* spp. inhabit Europe and Asia: (i) *P. oxyurus*, (ii) *P. slavaukrainii* sp. nov., (iii) *P. sphincteropharynx*, (iv) *P. skrjabini* and (v) *P. longicirratus*. Considering that *P. oxyurus* and *P. slavaukrainii* sp. nov. were discussed above, we opt to discuss only the remaining three species below.

*Psilochasmus sphincteropharynx* was described from the rock dove *Columba livia* Gmelin and the mallard. Oshmarin (1970) described the pharynx of this species as having a strongly developed muscular ring at its anterior end that appears sphincter-like. Unfortunately, subsequent authors, notably Yamaguti (1971), did not comment on this species. In our opinion, the unusual morphology of this species supports its validity, at least until new material is available.

*Psilochasmus skrjabini* was originally described from the ferruginous duck *Aythya nyroca* (Güldenstädt) in present day Azerbaijan (Gnedina 1946). Yamaguti (1958) synonymized *P. skrjabini* with *P. oxyurus*. Premvati (1969) and Mehra (1980) agreed with this action. However, Yamaguti (1971) considered *P. skrjabini* to be a valid species. Loos-Frank (1968) described *Psilochasmus aglyptorchis*Loos-Frank (1968) based on adults from an experimentally infected European herring gull *Larus argentatus* Pontoppidan in Germany. Yamaguti (1971) also considered *P. skrjabini* and *P. aglyptorchis* to be separate species. Both *P. skrjabini* and *P. aglyptorchis* have an extremely short esophagus with the cecal bifurcation immediately posterior to the pharynx, whereas other congeners have a well-developed esophagus with the cecal bifurcation near the anterior margin of the ventral sucker. We fail to find any meaningful features that support the status of *P. skrjabini* and *P. aglyptorchis* as separate species. Therefore, we consider *P. aglyptorchis* a junior synonym of *P. skrjabini*.

*Psilochasmus longicirratus* was originally described by Skrjabin (1913) based on specimens from a ferruginous duck in present day Kazakhstan. *Psilochasmus longirratus* and *P. oxyurus* were primarily distinguished based on the posterior margin of the cirrus-sac reaching the posterior margin of ovary in *P. longicirratus* but not in *P. oxyurus*. Stunkard and Dunihue (1931) considered this difference to not be substantial enough to separate these species and synonymized *P. longicirratus* with *P. oxyurus*. Premvati (1969) and Mehra (1980) maintained this synonymy, but Yamaguti (1971) recognized *P. longicirratus* as a valid species. Some of our specimens of *P. oxyurus* have a cirrus-sac that reaches posteriorly to the level of ovary, whereas in others it is positioned somewhat anterior to the ovary; this feature alone is not suitable for separating species. However, the eggs of *P. longicirratus* (116–124) are noticeably longer than those of *P. oxyurus* (79–99 in the present material; 82–100 in Braun (1902)). We agree with Yamaguti (1971) that *P. longicirratus* is likely a valid species.

Several other species have been described, although most certainly represent *P. oxyurus* and/or *P. longicirratus*. *Psilochasmus japonicus* Ischii, 1935 was described from a ferruginous duck in Japan and has long been considered a synonym of *P. longicirratus* (Ischii 1935; Yamaguti 1939, 1971; Premvati 1969). Gupta (1957) described *Psilochasmus indicus* Gupta, 1957 based on a single specimen obtained from a ruddy shelduck *Tadorna ferruginea* (Pallas) in India. Premvati (1969) and Mehra (1980) considered *P. indicus* to be a synonym of *P. oxyurus*, whereas Yamaguti (1971) listed it as a valid species. *Psilochasmus indicus* and *P. oxyurus* were distinguished mainly based on the position of the genital pore in relation to the cecal bifurcation (anterior to it in *P. indicus* vs at the level of the cecal bifurcation in *P. oxyurus*). In addition, Gupta (1957) used the weakly-developed lobation of the testes in *P. indicus* to distinguish it from *P. oxyurus*. Our properly fixed and relaxed material of *P. oxyurus* exhibits different levels of testicular lobation (i.e., weak to strong). We do not consider the slight difference in genital pore position enough to separate the species and thus accept the synonymy of *P. indicus* with *P. oxyurus* indicated by Premvati (1969).

Jaiswal (1957) erected two species from India: *Psilochasmus alii* Jaiswal, 1957 from the knob-billed duck *Sarkidiornis melanotos* (Pennant) and *Psilochasmus megacetabulus* Jaiswal, 1957 from the Indian pond heron *Ardeola grayii* (Sykes). The original illustrations of both species suggest a poor state of the type-material. Jaiswal (1957) considered the eggs of *P. alii* (110–130) to be distinctly larger than *P. longicirratus* (116–124 long), which is certainly not true. The only feature that separates these species is that the cirrus-sac of *P. alii* only reaches the anterior margin of the ovary whereas in *P. longicirratus* the posterior margin of the cirrus-sac reaches the posterior margin of ovary. Mehra (1980) considered *P. alii* a synonym of *P. oxyurus*. However, we believe that this synonymization needs additional proof using quality specimens and DNA sequence data.

*Psilochasmus megacetabulus* was described based on a single specimen. Strangely, Jaiswal (1957) only differentiated *P. megacetabulus* from *P. alii*. Besides the rather large ventral sucker of *P. megacetabulus* (1,030 × 740), the morphology of this species suggests that it should be considered a synonym of *P. oxyurus*, as indicated by Mehra (1980). At the same time, the eggs of *P. megacetabulus* (98–116 long) are somewhat larger than *P. oxyurus* (79–99 in present material; 82–100 in Braun (1902)), but smaller than *P. longicirratus* (116–124). In part, the few differences in morphology may be the result of parasitism in an unusual host (an ardeid). All other descriptions of *P. oxyurus* and its synonyms are from anatids. Premvati (1969) considered *P. alii* and *P. megacetabulus* to be synonyms of *P. oxyurus*, whereas Yamaguti (1971) maintained both as distinct species. We agree with Premvati (1969) that *P. megacetabulus* is likely a synonym of *P. oxyurus*. At the same time, we synonymize *P. alii* with *P. longicirratus*.

*Psilochasmus singhi* Jaiswal & Humayun, 1971 was described based on two specimens from the lesser whistling duck *Dendrocygna javanica* (Horsfield) in India. Jaiswal and Humayun (1971) did not differentiate *P. singhi* from either *P. oxyurus* or *P. longicirratus*. The morphology of *P. singhi* is quite similar to both those species. The cirrus-sac in *P. singhi* is well anterior to the level of the ovary, whereas the egg length is exceptionally large (90–142). Unfortunately, it is impossible to determine whether measurements were limited to mature eggs. The large range may be due to the inclusion of measurements of immature or poorly positioned eggs. It cannot be entirely ruled out that *P. singhi* represents a distinct species, but it may well be a synonym of *P. longicirratus*. We consider *P. singhi* to be a *species inquirenda*.

### ﻿Key to species

Below we provide a new key to *Psilochasmus* spp. based on adult morphology. Measurements used in the keys are limited to the original descriptions and from specimens associated with DNA sequences. Unfortunately, there appear to be no clear morphological differences in adult worms that distinguish *P. oxyurus* and *P. agilis*. The only differences between these species is their distributions (Old vs New World) and DNA sequences (Table 3).

**Table:** 

1	Sphincter-like muscular ring at anterior end of pharynx present	***Psilochasmus sphincteropharynx* Oshmarin, 1970**
–	Sphincter-like muscular ring at anterior end of pharynx absent	**2**
2	Esophagus extremely short. Cecal bifurcation immediately posterior to pharynx	***Psilochasmus skrjabini* Gnedina, 1946**
–	Esophagus long. Cecal bifurcation near level of ventral sucker	**3**
3	Body with distinct lateral expansions posterior to level of ventral sucke	***Psilochasmus slavaukrainii* sp. nov.**
–	Body without distinct lateral expansion posterior to level of ventral sucker	**4**
4	Body 6 mm or greater. Oral sucker width to pharynx width ratio 1.1–1.5, mean 1.3	***Psilochasmus urbeni* sp. nov.**
–	Body 5 mm or less. Oral sucker width to pharynx width ratio greater than 1.6	**5**
5	Egg length greater than 110 µm	***Psilochasmus longicirratus* Skrjabin, 1913**
–	Eggs length 100 µm or smaller	**6**
6	Distributed in the Old World	***Psilochasmus oxyurus* (Creplin, 1825)**
–	Distributed in the New World	***Psilochasmus agilis* Travassos, 1921**

## ﻿Conclusions

The growing amount of molecular and morphological evidence provides proof that *Psilochasmus* is a diverse genus. Additional sequencing of *Psilochasmus* spp. from the Old World, notably from the Indian subcontinent, is needed to better assess the diversity of the genus and the interrelationships of its constituent species. Importantly, morphological descriptions associated with DNA sequences are needed to better understand both the potential morphological variation within *Psilochasmus* spp. and the relative diagnostic value of different morphological features.

## Supplementary Material

XML Treatment for Psilochasmus oxyurus

XML Treatment for Psilochasmus slavaukrainii

XML Treatment for Psilochasmus urbeni

## ﻿Additional information

### Use of AI

No use of AI was reported.

### Author ORCIDs

Tyler J. Achatz https://orcid.org/0000-0003-2472-4372

Sarah A. Orlofske https://orcid.org/0000-0002-6103-7005

Martin M. Montes https://orcid.org/0000-0002-7177-333X

Sara V. Brant https://orcid.org/0000-0003-4488-7566

Vasyl V. Tkach https://orcid.org/0000-0001-5084-7566

### Data availability

All of the data that support the findings of this study are available in the main text.
